# Prototyping a GNSS-Based Passive Radar for UAVs: An Instrument to Classify the Water Content Feature of Lands

**DOI:** 10.3390/s151128287

**Published:** 2015-11-10

**Authors:** Micaela Troglia Gamba, Gianluca Marucco, Marco Pini, Sabrina Ugazio, Emanuela Falletti, Letizia Lo Presti

**Affiliations:** 1 Istituto Superiore Mario Boella (ISMB), Via P.C. Boggio 61, 10138 Torino, Italy; E-Mails: marucco@ismb.it (G.M.); pini@ismb.it (M.P.); falletti@ismb.it (E.F.); 2 Politecnico di Torino - Corso Duca degli Abruzzi 24, 10129 Torino, Italy; E-Mails: sabrina.ugazio@polito.it (S.U.); letizia.lopresti@polito.it (L.L.P.)

**Keywords:** UAV, GNSS-reflectometry, GNSS bistatic radar, prototyping

## Abstract

Global Navigation Satellite Systems (GNSS) broadcast signals for positioning and navigation, which can be also employed for remote sensing applications. Indeed, the satellites of any GNSS can be seen as synchronized sources of electromagnetic radiation, and specific processing of the signals reflected back from the ground can be used to estimate the geophysical properties of the Earth’s surface. Several experiments have successfully demonstrated GNSS-reflectometry (GNSS-R), whereas new applications are continuously emerging and are presently under development, either from static or dynamic platforms. GNSS-R can be implemented at a low cost, primarily if small devices are mounted on-board unmanned aerial vehicles (UAVs), which today can be equipped with several types of sensors for environmental monitoring. So far, many instruments for GNSS-R have followed the GNSS bistatic radar architecture and consisted of custom GNSS receivers, often requiring a personal computer and bulky systems to store large amounts of data. This paper presents the development of a GNSS-based sensor for UAVs and small manned aircraft, used to classify lands according to their soil water content. The paper provides details on the design of the major hardware and software components, as well as the description of the results obtained through field tests.

## 1. Introduction

Over the past few decades, Global Navigation Satellite System (GNSS) signals have not been used solely for navigation purposes. Indeed, since satellites can be considered a passive source of radiation, GNSS signals have been used for remote sensing applications, which consist of the processing of GNSS signals reflected back from the ground. Such reflected signals can be used to characterize the Earth’s surface, because they have different characteristics from those of the signal directly received from the satellite, in terms of delay, Doppler shift, power strength and polarization. These differences depend on the geophysical properties of the scattering surface; therefore, they potentially carry information about the surface geophysics.

GNSS signals are broadcast over the L-band, and many experiments have successfully demonstrated GNSS-reflectometry (GNSS-R) for the remote sensing of land and ocean surfaces [1] using the GPS L1 at 1575.42 MHz. Wind retrieval and altimetry, mainly from static platforms [2], are the most consolidated applications, while new employments, such as soil moisture sensing, ice monitoring, water level and snow thickness measurements [3], are continuously emerging and are presently under development. More recently, the joint use of GNSS-R data and other sensors, such as optical, infrared, thermal and microwave radiometers, turns out to be promising for accurate soil moisture estimation [4] and sea surface salinity retrieval [5,6]. In addition to better environmental monitoring, it is expected that new GNSS-R data will represent valuable inputs to numerical weather prediction (NWP) systems. Although today’s NWPs are capable of predicting many meteorological events, their accuracy is sometimes poor or they have an insufficient lead-time to initiate actions aimed at protecting life and property. For instance, uncertainty in present meteorological forecasts and the lack of integration of currently scattered monitoring networks represent a bottleneck for flood and drought risk assessment at local and regional scales. GNSS-R can be a means to provide additional data at low cost, mainly if new GNSS-R devices are mounted on-board unmanned aerial vehicles (UAVs). Today, UAVs offer a broad range of solutions for many civilian applications and can be equipped with several types of sensors for environmental monitoring; some UAVs are also more cost effective with respect to manned light aircraft. Until now, many instruments for GNSS-R have been proposed, and several algorithms have been developed for the estimate of geophysical properties of the scattering materials (e.g., [1,7]) and for altimetry (e.g., [2,3,8,9]). The hardware of traditional GNSS-based passive radars consists of custom GNSS receivers, based on application-specific integrated circuits (ASIC) or field programmable gate arrays (FPGA). In most cases, they require a personal computer (PC) and sufficient memory to store a large amount of data [10,11]. GNSS-based passive radars use a right-hand circular polarized (RHCP) antenna pointing toward the zenith for the reception of the direct signals from satellites and a second left-hand circular polarized (LHCP) antenna pointing towards the nadir for the reception of the reflected signals. Some devices (e.g., [12]) have been designed to collect the LHCP-only reflected component, because most of the reflected power has this polarization. More advanced versions (e.g., [13]) enable the reception of both LHCP and RHCP polarizations, because even weak RHCP reflected signals carry valuable information for precise measurements, like for the estimate of the soil moisture. It is worth noticing that other configurations making simultaneous use of horizontally- and vertically-polarized antennas, such as in [14], are possible, but their use on-board a small UAV would pose several mounting and reception issues.

The main drawback of many GNSS-based bistatic radars proposed so far is the heavy and bulky set up, which prevents the use of such devices on-board small and light UAVs. To overcome this problem, some researchers spent effort to design more compact and portable instruments. For example, Esterhuizen [15] proposed a software receiver over a Nano-ITX single board computer combined with two radio frequency (RF) front-ends featuring a common clock, connected to a universal serial bus (USB) bridge for high-speed data transfer. In [16,17], a prototype of an FPGA-based real-time GPS reflectometer is presented, which computes the full two-dimensional delay Doppler maps every 1 ms and performs coherent and incoherent averaging. Other remarkable examples are the designs of Starlab: Oceanpal^®^ [12] and the SAMGNSS reflectometer [18,19] are two instruments that they developed. While the former collects the LHCP reflected GNSS signals from the sea surface, the latter enables the reception of both polarization components of the reflected signal for soil moisture retrieval [13,18,19,20,21].

This paper presents the design and prototyping of a GNSS-based bistatic radar for small UAVs to be employed in environmental monitoring campaigns, for the water content classification of land and for the detection of water surfaces. Section 2 introduces the major requirements that guided the first phase of the design: among all, the light weight and the reduced size of the sensor, as well as the need for a GNSS antenna able to receive the LHCP and RHCP components of the reflected signals over two separate channels. Section 3 provides an overview of the hardware architecture, with a functional block diagram and the layout of the components, which were integrated into a case with an airfoil shape. It also explains some details of the software running on the microprocessor that controls the overall system, developed under a software radio paradigm to introduce flexibility. The remarkable hardware feature consists of the capability to simultaneously collect both polarizations’ data streams, synchronized with the same clock. This makes the proposed sensor different with respect to the reflectometer presented in [18], which, on the contrary, switches among the reflected RHCP and LHCP RF signals and, therefore, processes them in a sequential way. Although the focus of this work is not on the reflectometry data post-processing, but on the GNSS sensor design and implementation, Section 4 briefly discusses the background of such a discipline to give better evidence of the overall process that starts from GNSS measurements and ends with moisture-related information. Thus, Section 5 proceeds with the description of some results obtained in the field with a small aircraft. Finally, Section 6 concludes the paper with some open issues and the expected developments and exploitation of this work.

## 2. Rationale and Requirements

The objective of this work is the design and prototyping of the on-board sensor to collect measurements of GNSS reflected signals suitable to enable the estimate of some soil parameters, in particular the soil moisture, using the GNSS sensor mounted on-board a small, possibly unmanned, aircraft. To implement the radar capabilities, the direct signal coming from the satellite is received for positioning purposes, in order to evaluate and geo-reference the specular reflection point on the ground, as described in Section 4; furthermore, the characteristics of the direct signal are used as a reference for the processing of the reflected one, to enable the remote sensing of the soil features.

**Figure 1:**
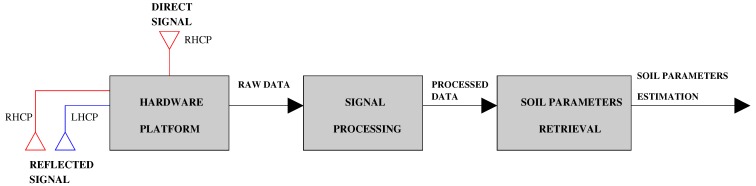
High-level information flow and the three layers of the prototype design.

The design flow of the on-board sensor has been distributed over three main layers, depicted in Figure 1:

- the hardware platform,
- the GNSS signal processing and
- the signal processing for soil parameter retrieval.

The first step is the definition of the hardware architecture, *i.e.*, the RF front-end, the microprocessor board and the antennas. Then, the design of proper GNSS signal processing techniques follows, to detect and estimate the relative delay and the amplitude of the reflected GNSS signals. The major concern at this stage is the extreme weakness of the reflected signals, which may lose around 13 dB for the LHCP and 23 dB for the RHCP with respect to the direct signal [18], received at a nominal power on the order of −160 dBW. Finally, proper remote sensing algorithms post-process the raw GNSS data.

The focus of this paper is explicitly on the hardware architecture of the prototype. For this reason, only a few details are given about the signal processing and soil parameter retrieval algorithms; the interested reader may refer to [22,23]. Nonetheless, Section 5 shows some examples of the results obtained from processing the data recorded by the prototype, during one of the test flights.

The design of the GNSS-based bistatic radar was guided by a set of requirements dictated by the target application. The subset of functional requirements applicable to the hardware platform discussed here is constituted by six propositions:

**Advanced antenna configuration.**The sensor shall be able to handle three antennas, implying three RF chains and three digital streams, as depicted in Figure 1.

**Storage capability.** The sensor shall be able to store raw measurements, observed during a flight mission.

**Direct and reflected signals’ synchronization.** The three signal streams (one direct, two reflected) shall be synchronized in sampling, storage and processing. Since the reflected signals are in general very weak, their processing can obtain significant benefits if aided by the direct signal, but aiding procedures require the synchronization among the three streams.

**Flexibility.** The radar shall be programmable and reconfigurable, at least in terms of the receiving bandwidth, signal conditioning and digital signal processing parameters (e.g., the number of correlation points).

**Processing capability.** The radar should be equipped with enough computing resources to allow the implementation of some on-board digital signal processing algorithms.

**Size and weight.** The sensor must be lightweight, *i.e.*, <3 kg, and small, *i.e.*, ≤200 mm × 250 mm × 250 mm (length × width × height), to be mounted on-board UAVs and light aircraft.

## 3. Prototype Design

From the high level application requirements listed above and from the indications received by the UAV manufacturer during the phases of development, the fundamental features of the sensor’s hardware platform were derived. They are presented in the next subsections, organized by hardware components, software components and functional validation.

### 3.1. Hardware Components

The essential hardware components of the on-board sensor are:

1. the GNSS antennas,
2. the commercial off-the-shelf (COTS) RF front-ends (FEs) and
3. the digital signal processing (DSP) stage.

**Figure 2:**
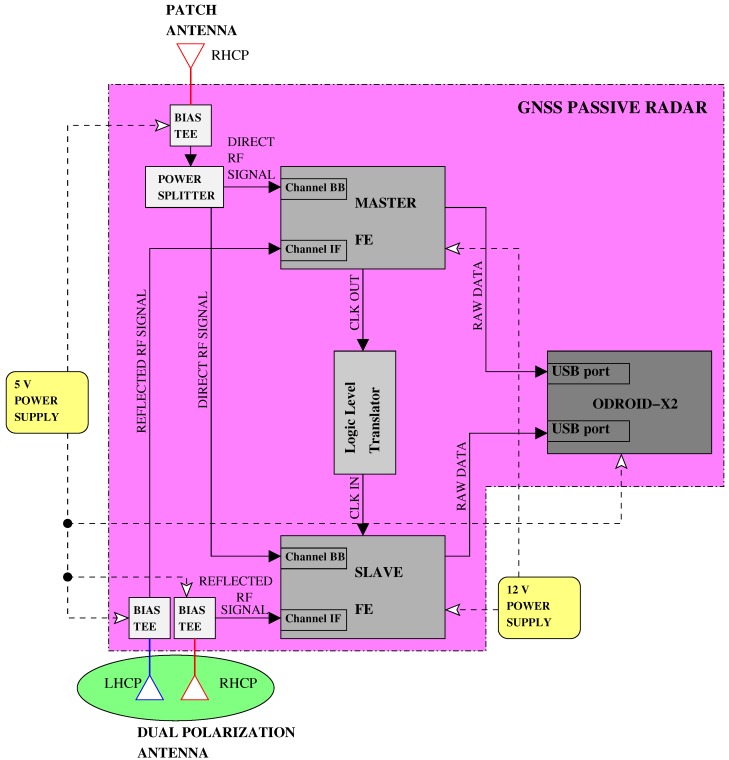
Hardware scheme of the sensor.

Figure 2 shows the functional block diagram of the sensor, with the major hardware components highlighted and their connections.

While a conventional low-cost hemispherical GNSS L1 RHCP patch antenna, properly mounted to point to the zenith and normally available on-board, is enough to receive the direct GNSS signals, the reflected ones require an *ad hoc* antenna oriented toward the nadir. Since one of our purposes was the reception of the reflected signals with both polarizations (LHCP + RHCP), we preferred a single dual-polarization antenna instead of two separate single-polarization ones, in order to limit weight and volume. However, very stringent requirements were set against the level of cross-polarization isolation, which represents a measure of the cross-talk between the two nominal polarizations. The work in [18] suggests a value lower than −24 dB, in particular for the measurement of the very weak RHCP component of the reflected signals against the stronger LHCP component. Unfortunately, commercial products typically do not meet both requirements of weight and cross-polarization isolation. Nonetheless, we decided to adopt the dual-polarization L1/L2 GNSS Antcom antenna 1G1215RL-PP-XS-X RevA [24], whose cross-polarization isolation declared by the manufacturer is −17 dB; despite its suboptimal performance in polarization separation to perform precise GNSS-R polarimetric measurements [18], it is light, small and has a quite flat profile. Another custom Antcom device based on the G8ANT-52A4SC1-RL model, whose cross-polarization rejection specification was set at −24 dB, showed RF compatibility problems with the front-end and cross-polarization issues, making its use more difficult and even having lesser performance.

The second stage of the hardware platform is the RF front-end. It includes the stages of signal conditioning, RF down conversion, filtering and analog-to-digital conversion (ADC). The number of RF chains in the FE must be equal to the number of separate signals and polarizations: one RF chain is employed for the direct signal and connected to the zenith-pointing antenna, while two RF chains are devoted to the two LHC- and RHC-polarized reflected signals and connected to the two ports of the nadir-pointing antenna. The same clock reference must be distributed on the three chains. To implement this setup, two FEs of the “Stereo” family commercialized by Nottingham Scientific Ltd (NSL) were selected [25], configured in a master/slave architecture. Each FE embeds two full and synchronized receiving chains, implemented in two separate chipsets: the MAX2769B, covering the GNSS upper L-band and indicated as L1, and the MAX2112, covering both the upper and lower L-bands and indicated as LB. In Figure 2, we identified the two chains as “Channel IF” and “Channel BB” respectively, to indicate the different down conversion schemes applied in the two chains. The FE contains one shared clock (TXC 26 MHz TCXO) with interfaces for alternative oscillators and external frequency input. Slaving two FEs to the same clock guarantees four synchronized channels. To do this operation, a logic level translator, from low voltage positive emitter coupled logic (LVPECL) to low voltage complementary metal oxide semiconductor (LVCMOS) levels was specifically designed and manufactured as a printed circuit board (PCB). In Figure 2, the exact connection between antennas and FE chains is indicated: the direct signal is split and sent to Channel BB of both the FEs as a reference, while the reflected LHCP and RHCP are connected to the Channel IF of the master and slave boards, respectively.

Finally, a DSP stage is necessary to process the digital data after the ADC. For our purposes, the software-defined radio (SDR) is the preferred technology over other solutions like FPGA or ASIC-based platforms, thanks to its flexibility, re-configurability and reduced development time in the prototype integration. The DSP stage was required to support a memory of at least some tens of GBytes, e.g., in a secure digital (SD) card or an embedded multimedia card (eMMC) card, for fast storage of the raw data produced during a flight mission. A number of I/O USB ports was also necessary to handle digitalized data streams and to give access to the sensor configuration parameters. The chosen platform is the Open-Android (ODROID)-X2 [26]. It is an open development 1.7 GHz ARM Cortex-A9 Quad Core platform with 2 GB RAM memory and PC-like performance. The ODROID-X2 was one of the most powerful boards available on the market at the time the activity began. It provides 2 GB RAM memory and a number of peripherals, like a high-definition multimedia interface (HDMI) monitor connector and six USB ports. It is able to support the input from the two FEs streams, thanks to the real-time management of two USB ports and the fast memory storage. This board hosts an Ubuntu Linaro Operative System (OS) distribution, booting from a 64 GB eMMC. In the current version, the sensor serves as data grabber: data are received, sampled and stored in the memory. Further developments will address the implementation of some more advanced processing directly on-board.

As indicated in Figure 2, a power supply of 5 V is employed for the ODROID-X2 and all of the antennas, while 12 V is used for the FEs in order to guarantee a proper functioning of the device, in particular the stability of the master clock. The bias-tees (BTs) allow a stable power supply to the antennas, while decoupling the DC from the RF signal entering the FE.

A summary of the fundamental hardware components of the radar prototype is reported in Table 1.

**Table 1:** Summary of the selected principal hardware components. eMMC, embedded multimedia card.

Hardware Component	Selected Device
Antenna (towards zenith):	Aircraft’s hemispherical L1 patch
Antenna (toward nadir):	Antcom dual-polarization L1/L2 1G1215RL-PP-XS-X RevA
RF front-end:	NSL Stereo (2 boards mutually synchronized)
DSP (*μ*-processor board):	ODROID-X2, 1.7 GHz ARM Cortex-A9 Quad Core platform, 2 GB RAM
Memory:	64 GB eMMC

### 3.2. Hardware Assembly

**Figure 3:**
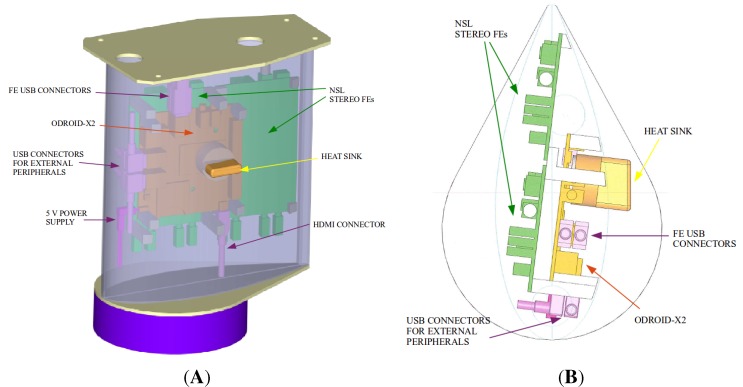
Three-quarter (**A**) and top (**B**) views of the sensor case 3D and 2D CADs, with the main internal components visible.

After the definition of the functional architecture, the system components were assembled inside a proper case. The authors already showed a preliminary assembled prototype in [27], but such a configuration implied a parallelepiped-shaped case, which was not the best in terms of aerodynamic performance. For this reason, the final sensor case was designed *ad hoc* in carbon fiber with a neutral wing profile, and the internal components were mounted accordingly. Some computer-aided design (CAD) views are reported in Figure 3. In particular, Figure 3A depicts the carbon fiber case in light violet, double-ended with two aluminum plates in grey. The bottom plate serves as the support for the nadir-oriented antenna, depicted by the dark violet cylindrical disk, while the top lid is designed to be screwed to a rectangular plate, called the trolley unit, which connects the sensor mechanically and electrically to the aircraft body. The trolley is specifically designed to be hosted in a structure (bay) composed of two rails and fastened externally to the lower part of the aircraft, for rapid boarding of the sensors. The bay can host 3–4 sensor carts boarded with a “plug-and-play” mechanism. The yellow object, which stands out from the case, acts as heat sink of the internal parts: in fact, it terminates with a cylindrical part directly in contact with the microprocessor of the ODROID-X2 board, depicted in orange. The two FEs in green are just behind the ODROID-X2. All of the boards are attached to the internal faces of the case by means of small resin supports. The chosen airfoil is better visualized in Figure 3B, where it is possible to observe how the internal components are placed, in order to optimize the available space.

**Figure 4:**
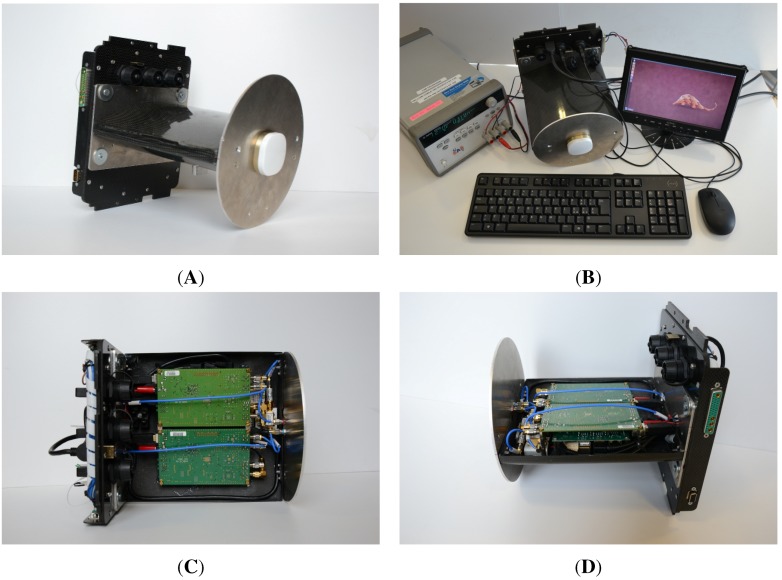
Photos of the sensor prototype, closed in its carbon fiber case (**A**), opened (**C**,**D**) showing internal components and equipped with a keyboard, mouse, HDMI monitor and power supply for the in laboratory tests (**B**).

Once the carbon fiber case has been manufactured, the assembly of the sensor was completed. Figure 4 shows some pictures of the final version of the prototype. In particular, the carbon fiber case is well visible in Figure 4A,B: it is screwed to the nadir-oriented antenna at the bottom side, by means of a circular aluminum plate with the function of both support and ground plane, and to the trolley unit at the top side. Figure 4B shows the sensor under lab testing connected to an HDMI monitor, mouse, keyboard and external power supply. The case has been specifically designed in such a way as to be easily opened, for fast checks and maintenance service, as illustrated in Figure 4C,D, where internal components and connections are visible.

Designed and assembled in such a way, the sensor prototype dimensions resulted in being 200 mm ×200 mm ×250 mm (length × width × height) with a weight of less than 3 kg, including the nadir-pointing antenna with its ground plane, so as to be sufficiently light and compact to be mounted on-board UAVs and small aircraft. In particular, the target UAV belongs to the civilian category of remotely-controlled (RC) light UAVs, or more in general, light unmanned aircraft systems (UASs), with a maximum take-off mass (MTOM), including the fuel, of less than 150 kg and an autonomy of 1–2 h. Additionally, the manned airplane is a two-seat ultralight aircraft with fixed wings.

### 3.3. Software Components

As said in Section 3.1, the ODROID-X2 has PC-like performance: it features several peripherals and hosts an Ubuntu Linaro Operative System (OS). Consequently, the entire development work was done directly on the target platform, with no need for another machine for cross-compiling. Nevertheless, the implementation of the master-slave configuration required not only the integration of the logic level translator, described in Section 3.1, at the hardware level, but also some additional work at the software level: the original FE drivers required to be modified and recompiled on the target platform, in order to manage the connection of the two FEs unambiguously.

Software components include routines for FE configuration, usage modes and grabbing functionality. Thanks to the flexibility of the selected FEs, based on the SDR paradigm, the user is allowed to create his or her own setup, configuring the parameters listed in the first column of Table 2. In our setup, the parameter configuration is the same for both FEs in the sensor. In order to manage two USB data streams, the default sampling frequency chosen for the prototype was the lowest possible permitted by the manufacturer; this choice allows for storing more than 30 min of data on the ODROID-X2 eMMC, which is sufficient for our purposes.

**Table 2:** Configuration options for the RF front-ends. Channel IF and Channel BB are the two RF chains of each front-end. The rightmost column contains default values used during the tests. The admissible ranges are derived from the examples presented in the Stereo front-end (FE) user manual and have not been completely tested by the authors.

Configurable Parameter	Admissible Range	Default Value
Sampling frequency	13 ÷ 40 MHz	13 MHz
Channel IF, carrier frequency	{L1, E1, G1}	1575.42 MHz
Channel IF, intermediate frequency	Not specified	3.55 MHz
Channel IF, double-sided bandwidth	2 ÷ 9.66 MHz	4.2 MHz
Channel BB, carrier frequency	{L1, E1, G1, L2, G2, L5, E5a, E5b}	1575.42 MHz
Channel BB, intermediate frequency	0 MHz	0 MHz
Channel BB, single-sided bandwidth	1.39 ÷ 10.09 MHz	4.0 MHz
Channel BB, filter gain	0 ÷ 15 dB	6 dB

The sensor can be used in two modes, based on the number of signals the user desires to process:

1. Basic mode: direct channel + one LHCP reflected channel (only the master FE enabled);
2. Advanced mode: direct channel + two reflected channels (LHCP and RHCP).

Each mode is implemented via software, using proper shell scripts for the FE configuration, which are executed as startup applications. In this way, at power up, the ODROID-X2 automatically boots the OS, configures the FEs based on one of the above-described usage modes and launches the data grabbing, which uses the eMMC module as the storage unit. The start and stop commands and the duration of data grabbing are parameters to be defined based on the flight plan. Note that, with a 13 MHz sampling frequency, the two modes have a different impact on the necessary amount of memory: the advanced configuration requires 1.56 GB/min, allowing one to save more than 30 min of data, while the basic configuration halves the rate, thus doubling the total amount of storable data. Since the raw data are stored to memory, ready for off-line processing, the sensor is fully enabled for all of the available GNSS signals, and the information of all visible satellites is fully preserved: this approach facilitates a thorough validation of the prototype and an accurate interpretation of the soil parameter estimation.

### 3.4. Functional Tests for the Validation of the Sensor

For the functional validation of the prototype, an intensive test campaign was conducted in the laboratory. Such tests were divided into two categories: first, we validated the sensor in a controlled environment, generating GNSS signals through a hardware signal generator; then, we tested the signal conditioning with live GNSS signals.

**Figure 5:**
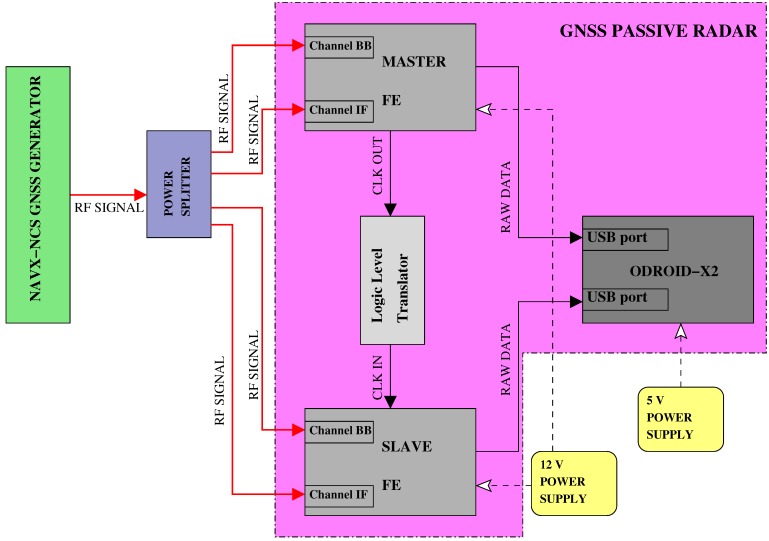
In-laboratory test setup with the simulated scenario.

**Figure 6:**
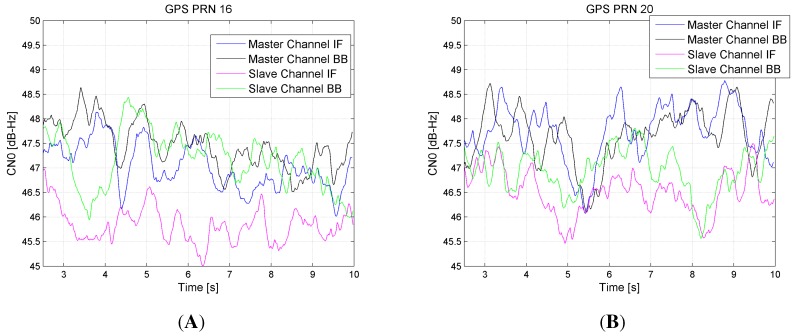
$C/N_{0}$ evaluation for PRN16 (**A**) and PRN 20 (**B**), obtained during a test in a simulated scenario.

First, we were able to verify all hardware components, from RF to IF, as well as the software routines implementing the usage modes and the grabbing functionality. The use of a professional GNSS hardware generator [28] allowed for excluding effects due to phenomena related to a real environment, such as multipath and interferers. Several tests were performed on each single RF receiving chain of the two FEs, which were first validated separately. Then, all of the channels were tested simultaneously, with the sensor configured such that the master FE provided the reference clock to the slave one. The same GNSS signal was split and sent to the four channels by means of a four-way power splitter, as reported in the simplified scheme of Figure 5. The signals at the RF input were digitalized, and the samples were stored in the ODROID-X2 eMMC memory, then post-processed by a software receiver. Several test metrics were considered in the analysis and validation process: the power spectral density (PSD) of the digitalized signal, the amplitude of the main peak of the cross ambiguity function (CAF) computed during the acquisition of the GNSS signals, the quality of the tracking loop lock through the mean and variance of the correlators and the estimate of the carrier-to-noise power density ratio ($C/N_{0}$). The sensor successfully passed all of the tests and demonstrated the ability to process the signal properly in all cases. The FEs resulted in being well synchronized through the master-slave configuration, whereas the ODROID-X2 was able to handle the two FEs’ streams, thanks to the real-time management of two USB ports and the fast memory storage. The post-processing analysis revealed that the receiver is able to successfully acquire and track all generated satellite signals in all performed tests. As an example, Figure 6 shows the estimated $C/N_{0}$ for two tracked satellites, processing the streams of samples at the output of the four channels. The $C/N_{0}$ is estimated at the tracking loop stage and provides a valid measure of the quality of the received signal [29]. Looking at Figure 6, it can be noticed that the values of $C/N_{0}$ measured on samples out of the slave FE after the initial transient (*i.e.*, the pink line for the first channel and green for the second) are on average 1 dB lower with respect to the $C/N_{0}$ estimated on the corresponding chains of the master FE, depicted in blue and black, respectively. The reason for this small power loss is explained considering that the slave FE receives the clock from the master, via a logic translator circuit, which introduces noise to the reference signals of the slave board.

**Figure 7:**
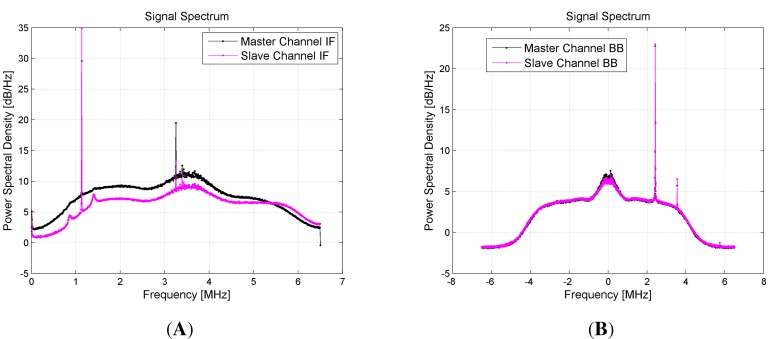
Comparison of the estimated power spectral densities of the signals at the two channels of both FEs, generated through a hardware signal generator and supplied to the FEs input ports through a wired RF connection. (**A**) Channel IF; (**B**) Channel BB.

In Figure 7, another example of the in-lab test results is reported. Here, the estimated PSDs of the digitalized signals entering the FEs are shown: Channel IF signals in Figure 7A and Channel BB signals in Figure 7B. In particular, the black plot represents the master spectrum, while the pink one is the slave spectrum. The GPS signal is strong and well evident in the bandwidth center. From these results, a strong interferer at ±2.4 MHz respectively for IF=0 Hz (BB) and IF=3.55 MHz (IF) appears. This is probably due the FE clock, but being far from the GPS main lobe, no performance degradation is produced in the acquisition and tracking loop.

In the second part of the validation, we processed real signals received from the antennas, although limited to live RHCP signals, because it was not possible to replicated in-lab LHCP reflected signals. Anyway, from a functional perspective, such tests were necessary to check the performance of the FEs when connected to the antennas and to detect any distortions on the received signals. The test setup corresponds to the scheme of Figure 2, with the only difference that the dual-polarization antenna is pointed towards the sky, as well as the antenna for the reception of direct signals, and only the RHCP channel is evaluated. The results of these tests, using the same performance metrics mentioned before, allowed for an accurate calibration of the hardware platform parameters, in particular at the signal conditioning stage, to find the best match between antennas and RF receiving stages. Using the calibrated setup, the sensor showed good performance, and no anomalies on the received signals were detected.

## 4. Soil Moisture Retrieval from Reflection Measurements: A Background on the Discipline

In this section, we briefly review the basic principles of the soil moisture estimate using GNSS reflectometry. A review of the theoretical background of this discipline is necessary in order to clearly motivate the implementation choices made in the prototype design.

In order to quantitatively estimate the soil moisture, the soil dielectric constant has to be evaluated, applying models that take into account the soil characteristics, including in particular the soil composition [18,30,31]. Different methods exist, which either exploit the LHCP reflected signal only or both the LHCP and the RHCP reflections. The soil dielectric constant depends in particular on two parameters related to the GNSS signal: the soil reflection index and the incidence angle (which directly depends on the satellite elevation angle).

In the following paragraphs, the main equations are provided, explaining the physical principles at the basis of the soil moisture retrieval through the measurement of the reflected GNSS signal. The principle is that the soil dielectric constant changes depending on the soil water content, which has an impact on the soil reflectivity properties; it is highly dependent on the soil composition, as well. In particular, the more the soil is moist, the less an incising electromagnetic wave penetrates it in depth, which translates into a higher reflected power [32]. However, a role is played also by the surface roughness, which decreases the specular reflected power.

The two basic principles summarized hereafter exploit, on the one hand, the LHCP measurements only, on the other one, the joint processing of the LHCP and RHCP measurements.

### 4.1. LHCP-Based Soil Moisture Retrieval

In the simplified hypothesis of a specular reflection, the soil reflection index can be estimated from the ratio between the reflected and the directly incident signal power. This quantity can be evaluated as the ratio of the estimated signal-to-noise ratios (SNR) on the reflected and on the direct channel, respectively. In this way, the differences in the hardware receiving chains of the reflected and the direct signals can be compensated. Once the soil reflection index is known, the dielectric constant can be retrieved. Thus, in order to estimate the soil dielectric constant from the GNSS reflection measurement, it is needed to *a priori* know the satellite elevation (incidence angle) and to have at least a rough knowledge of the soil composition. Then, the reflection index must be estimated, which is a function of the reflected power percentage after the incidence. In what follows, the concepts explained so far will be provided in formulas.

If the reflecting surface can be well approximated as a perfectly smooth surface, then a specular reflection can be assumed. In such a case, neglecting any surface roughness and, therefore, any non-coherent components in the reflection, the reflected GNSS signals results in being mainly LHCP, in particular considering the satellites with close to the zenith elevation. For the direct signal propagating in free space, the SNR is directly proportional to the transmitted power $P^{t}$ and the transmitter gain $G^{t}$, the receiver gain for the direct signal chain $G_{dir}^{r}$, the signal wave-length *λ* and the processing gain $G_{D}$. Then, it is inversely proportional to the transmitter-receiver distance *R* and to the noise power, which, for the direct signal receiving chain, is indicated as $P_{N,dir}^{r}$. Thus:

(1) $$SNR_{dir}=\frac{P^{t}G^{t}}{4\pi R^{2}}\cdot \frac{G_{dir}^{r}\lambda^{2}G_{D}}{4\pi P_{N,dir}^{r}}$$

Similarly, for the LHCP reflected channel, the SNR can be expressed as the power ratio between the reflected signal and the noise related to that channel. It can be written replacing in Equation (1) $G_{dir}^{r}$ with the receiver gain through the appropriate chain $G_{refl,l}^{r}$ and the traveled distance *R* with the sum of the distances from the satellite to the reflection point ($R_{A}$) and back to the receiver ($R_{B}$). Furthermore, it is necessary to account for the additional path loss due to the reflection, which can be written as $\frac{1}{4}{\left(|\Gamma_{vv}\left|+\right|\Gamma_{hh}|\right)}^{2}$ [33], where $|\Gamma_{vv}|$ and $|\Gamma_{hh}|$ are the reflection indexes for the vertical and the horizontal polarizations, respectively, which combine together in the case of circular polarization. The noise power in the reflected signal chain is $P_{N,l}^{r}$. Thus:

(2) $$SNR_{refl,l}=\frac{1}{4}\frac{P^{t}G^{t}}{4\pi {\left(R_{A}+R_{B}\right)}^{2}}\cdot \frac{G_{refl,l}^{r}\lambda^{2}G_{D}}{4\pi P_{N,l}^{r}}\cdot \left(\right|\Gamma_{vv}\left|+\right|\Gamma_{hh}{\left|\right)}^{2}$$

where the subscript *l* in $SNR_{refl,l}$ and $P_{N,l}^{r}$ refers to the LHCP reflected polarization.

As said above, the reflection index is a function of both the reflecting surface characteristics and the incidence angle; therefore, it can be expressed as a function of the soil dielectric constant $\epsilon_{r}$ and the satellite elevation angle *θ*:

(3) $$\Gamma_{vv}\left(\epsilon_{r},\theta \right)=\frac{\sin\theta -\sqrt{\epsilon_{r}-\cos^{2}\theta }}{\sin\theta +\sqrt{\epsilon_{r}-\cos^{2}\theta }}$$

(4) $$\Gamma_{hh}\left(\epsilon_{r},\theta \right)=\frac{\epsilon_{r}\sin\theta -\sqrt{\epsilon_{r}-\cos^{2}\theta }}{\epsilon_{r}\sin\theta +\sqrt{\epsilon_{r}-\cos^{2}\theta }}$$

In order to evaluate the soil dielectric constant, from which the soil moisture can be retrieved if there is some knowledge of the soil composition, the ratio between the reflected SNR in Equation (2) and the direct SNR in Equation (1) is computed. It results in being:

(5) $$\frac{SNR_{refl,l}}{SNR_{dir}}=\frac{R^{2}}{{\left(R_{A}+R_{B}\right)}^{2}}\cdot {\left(\left|\Gamma_{vv}\left(\epsilon_{r},\theta \right)\right|+\left|\Gamma_{hh}\left(\epsilon_{r},\theta \right)\right|\right)}^{2}\cdot C$$

where $C=\frac{G_{refl,l}^{r}}{P_{N,l}^{r}}\cdot \frac{P_{N,dir}^{r}}{G_{dir}^{r}}$ depends on the hardware differences in the receiving chains, mainly due to antennas and RF filtering gains. The actual value of *C* must be determined with a calibration.

One of the more robust ways to calibrate the system for soil moisture purposes is the on-water calibration, used for example in [34], through multiple over-water overflights. This is because the expected reflected power over water is well known given the incidence angle, while over the terrain the uncertainty is higher, due to the imperfect knowledge of the soil composition and its inherent dis-homogeneity. In order to have a more accurate calibration, a measurement campaign should be done *in situ* with other sensors (hygrometers), for different soil types in different moisture conditions. This would involve the need of performing measurements for a long time, in order to have reliable measurements, and to compare all of the obtained results with the other sensors in the terrain. However, for the application at hand, the on-water calibration is proven to be quite an effective low-cost solution [34].

After the calibration, the dielectric constant $\epsilon_{r}$ in Equation (5) is solvable via numerical routines, given the knowledge of *R*, $R_{A}$, $R_{B}$ and *θ*. It has to be noted that from Equation (5), only $|\epsilon_{r}|$ can be evaluated: in order to get the full soil moisture information, the real and imaginary parts of $\epsilon_{r}$ need to be separated, which is possible considering empirical dielectric models, such as the one proposed in [31].

### 4.2. LHCP + RHCP-Based Soil Moisture Retrieval

The retrieval algorithm described above is based on the assumptions of having a smooth reflection surface. Nonetheless, in order to better take into account the effects of the soil roughness, which makes the reflection different from specular, another approach is needed, which exploits the availability of both the LHCP and the RHCP SNR measurements.

The roughness of a surface impacts its capability of reflecting an incident electromagnetic field along a principal direction (reflection angle); this capability is typically quantified in terms of the so-called radar cross-section RCS [35]. The RCS of an object is in turn a function of: (i) the object dimensions and shape; (ii) the electromagnetic wave incident angle; and (iii) the reflecting material (through the so-called normalized radar cross-section (NRCS), $\sigma^{o}$). The NRCS is a function of the dielectric properties of the material and separates into a horizontal and a vertical polarization component.

For these reasons, we expect it to be possible to extract the dielectric constant of the soil by estimating the NRCS for the two circular polarizations of the reflected GNSS signals (it is well known that the two LH and RH circular polarizations of an electromagnetic wave can be written as combinations of the two linear polarizations) [33].

The SNR of the reflected signals, which was expressed in Equation (2) for the LHCP reflection, can be expressed for both the LHCP and the RHCP reflections as:

(6) $$\begin{array}{ccc} SNR_{refl,l} & = & \frac{G^{t}G_{refl,l}^{r}\lambda^{2}\left|\sigma_{lr}\right|}{{\left(4\pi \right)}^{3}{R_{A}}^{2}{R_{B}}^{2}}\frac{P^{t}}{P_{N,l}^{r}} \end{array}$$

(7) $$\begin{array}{ccc} SNR_{refl,r} & = & \frac{G^{t}G_{refl,r}^{r}\lambda^{2}\left|\sigma_{rr}\right|}{{\left(4\pi \right)}^{3}{R_{A}}^{2}{R_{B}}^{2}}\frac{P^{t}}{P_{N,r}^{r}} \end{array}$$

where the notation is the one adopted in Equation (2), while the parameters $\sigma_{ij}$ represent the RCS for the circular polarized components of the incident and the reflected waves, for which the subscripts are such that *i* indicates the polarization of the reflected signal and *j* indicates the polarization of the incident wave. Furthermore:

(8) $$\begin{array}{ccc} \sqrt{\sigma_{lr}} & = & \frac{\sqrt{A}}{2}\left(\sqrt{\sigma_{hh}^{o}}+\sqrt{\sigma_{vv}^{o}}\right) \end{array}$$

(9) $$\begin{array}{ccc} \sqrt{\sigma_{rr}} & = & \frac{\sqrt{A}}{2}\left(\sqrt{\sigma_{hh}^{o}}-\sqrt{\sigma_{vv}^{o}}\right) \end{array}$$

where $\sigma_{hh}^{o}$, $\sigma_{vv}^{o}$ are the horizontal and vertical polarization components of the NRCS and *A* is the total illuminated area, or glistening zone, which depends on the reflection geometry [22,35]. The NRCS is a key parameter in reflection theory: through its estimation, the characteristics of the reflecting surface can be retrieved, but to do that, a good model of the reflecting system is required. A detailed analysis of the NRCS and of the effects of the geometry (incidence angle) is available in [36], where also the soil inhomogeneity is taken into account. The more accurate the model applied is, the more accurate the soil estimate will be. However, at this stage of the work, a simple approximated model has been considered, without a thorough study of the soil characteristics, as for instance the soil roughness.

Combining Equations (6) and (7) with Equations (8) and (9), the ratio between Equations (6) and (7) can be written as:

(10) $$\frac{SNR_{refl,l}}{SNR_{refl,r}}=\frac{\left|\sqrt{\sigma_{hh}^{o}}+\sqrt{\sigma_{vv}^{o}}\right|}{\left|\sqrt{\sigma_{hh}^{o}}-\sqrt{\sigma_{vv}^{o}}\right|}\cdot C^{\prime }$$

where $C^{{}^{\prime }}=\frac{G_{refl,l}^{r}}{P_{N,l}^{r}}\cdot \frac{P_{N,r}^{r}}{G_{refl,r}^{r}}$ is a calibration constant similar to *C* in Equation (5). The parameters $\sigma_{hh}^{o}$ and $\sigma_{vv}^{o}$ are functions, in particular, of the soil dielectric constant and the incidence angle (the satellite elevation); it can be stated that:

(11) $$\left\{\begin{array}{c} \sigma_{hh}^{o}=f_{1}\left(\epsilon_{r},\theta \right)\\ \sigma_{vv}^{o}=f_{2}\left(\epsilon_{r},\theta \right) \end{array}\right.$$

The functions $f_{1}$ and $f_{2}$, can be described through proper scattering models that take into account various other physical parameters involved in the reflection phenomena, other than the satellite elevation angle *θ*, assumed to be known, and the dielectric constant $\epsilon_{r}$ to be estimated. Different scattering models have been proposed in the literature [31,35,37]; for instance, applying the so-called small perturbation method (SPM), the RCS components can be expressed as a function of $\epsilon_{r}$ and *θ* [35]. To do that, for simplicity, the variables $\alpha_{hh}$ and $\alpha_{vv}$ can be introduced as:

(12) $$\left\{\begin{array}{c} \left|\alpha_{hh}\right|=\sqrt{\sigma_{hh}^{o}}\\ \left|\alpha_{vv}\right|=\sqrt{\sigma_{vv}^{o}} \end{array}\right.$$

Then, the relationship between $\alpha_{hh}$, $\alpha_{vv}$ (*i.e.*, $\sigma_{hh}^{o}$, $\sigma_{vv}^{o}$) and $\epsilon_{r}$, *θ* can be expressed as follows [35]:

(13) $$\left\{\begin{array}{c} \alpha_{hh}=\frac{1-\epsilon_{r}}{{\left(\cos\theta +\sqrt{\epsilon_{r}-\sin^{2}\theta }\right)}^{2}}\\ \alpha_{vv}=\frac{\left(1-\epsilon_{r}\right)\left(\epsilon_{r}-\sin^{2}\theta -\epsilon_{r}\sin^{2}\theta \right)}{{\left(\epsilon_{r}\cos\theta +\sqrt{\epsilon_{r}-\sin^{2}\theta }\right)}^{2}} \end{array}\right.$$

Thus, combining the expressions in Equations (12) and (13) with Equation (10) and determining the value of $C^{{}^{\prime }}$ through the calibration phase, the dielectric constant $\epsilon_{r}$ can be numerically solved.

## 5. Signal Processing and Results of an In-Field Test

Several in-flight data collection campaigns have been executed in order to test the performance of the prototype in different configurations. The aim of the test campaigns was to demonstrate the capability of the prototype to provide the GNSS measurements necessary to implement a soil moisture retrieval algorithm, such as one of those mentioned in the previous section. In this section, we first review the signal processing principles at the basis of our project (Section 5.1), then some results of the reflectometry measurement campaign are shown (Section 5.2).

### 5.1. Signal Processing Principles

With the scope of implementing the reflectometry functionalities, a MATLAB^®^-based software receiver has been modified here to make it able to properly process the data from the four channels of the sensor. The principle of this architecture comes from the GPS software receiver described in [38], and it has been chosen for the many advantages that the software-defined paradigm includes, in particular for its flexibility. Particular attention was paid to the study of the algorithms to detect the reflected signal to cope with the major challenges presented by the reflected signals, namely the extremely low power and the very short phase coherence.

In fact, the reflected signal is not a single specular reflection from the so-called specular point, but it is the sum of several contributions (scattering) from a reflecting area, namely the glistening zone, whose size depends on the incidence angle, the receiver altitude and the surface roughness (models exist that allow one to find a suitable approximation).

This scattering effect causes a much shorter phase coherence compared to the direct signal, in particular in dynamic environments, such as in flight; for this reason, an open-loop strategy is in general advisable to detect the reflections.

Furthermore, the scattering effect, reducing the reflected power reaching the nadir-pointing antenna, worsens for lower incident angles, in particular for the LHCP components. Thus, although Equation (5) takes the incidence angle into account, the accuracy of the estimate decreases when the satellite elevation reduces [34]: the SNR diminishes, meaning that the impact of the noise becomes heavier on the measurement. For this reason, the surface scattering effect needs to be mitigated in order to estimate the actual reflected signal power; this can be done by averaging over time the measurements.

Furthermore, in order to detect a low-power signal, the integration time needs to be increased as much as possible, even if this means getting lower spatial resolution on the measurements. However, the coherent integration time cannot be longer than the signal coherence interval; therefore, a trade-off solution needs to be found. A key strategy introduced in the software scheme is the channel aiding, which means that information from the direct signal processing is exploited to detect the reflected signal, since the Doppler frequency of the two signals is expected to differ by only a few tenths of Hertz, and the delay is expected to be within an interval depending on the satellite elevation and the aircraft altitude.

The effects of the secondary multipath are neglected here. Concerning the reflected signal, the interest is on the principal reflection; it might occur that some unexpected (and undesired) reflections from some targets, including buildings, are received together with the reflection from the considered reflection point on the terrain, as a multipath signal. Such cases are not predictable, but they are expected to be rare, and such an error is considered acceptable for this kind of application. Concerning the direct signal, undesired multipath signals may occur due to the reflections from the aircraft or the UAV. However, given the small dimensions of the aircraft on which the sensor is designed to be mounted and given the position of the antenna on the wing, the multipath effects are expected not to be significant with respect to the noise [23]. Anyway, a better analysis of the antenna gain together with the multipath effects should be included in the future developments of the sensor.

### 5.2. Test Campaign Results

**Figure 8:**
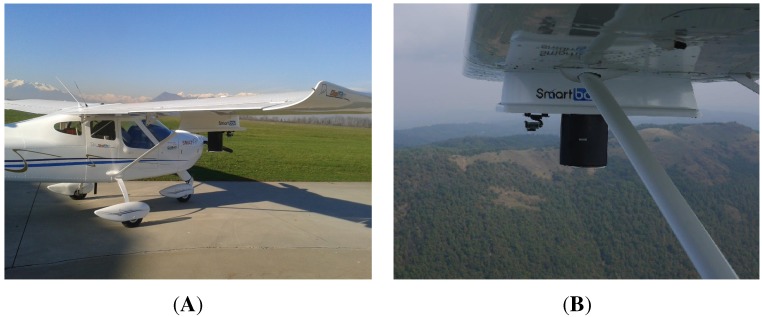
Sensor prototype mounted on the manned Digisky Tecnam P92 aircraft ready to take off (**A**) and during the flight (**B**).

**Figure 9:**
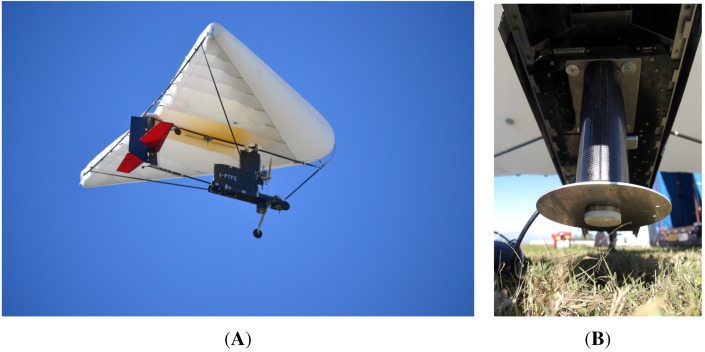
Sensor prototype mounted on the Nimbus C-Fly UAV during the flight (**A**) and on the ground after landing (**B**).

Some in-flight tests were executed to assess the prototype performance. The sensor was mounted on two different platforms, as shown in Figure 8 and Figure 9: a manned ultra-light aircraft (Digisky’s Tecnam P92) and a UAV (Nimbus’ CFly). Some results are shown here from a flight test with the P92 aircraft, which flew over a countryside nearby Turin (Italy), also overflying two small lakes (the Avigliana lakes). The overflown area has been chosen because it includes test scenarios of interest. In fact, this area is in the countryside north of Turin, not far from the airport from where the aircraft used for the tests can take off and land, and it includes water basins, such as lakes and rivers. Moreover, a swampy area is present around the lakes, which looks particularly interesting in the framework of this work, since the evaluation of the soil moisture is the main goal. In that region, different cultivated areas are also present, which are interesting from the perspective of a future agriculture application. Forest zones are present, as well, which are characterized by weaker reflections, due to the higher scattering effects; on this topic, several studies have been presented in the literature to address the analysis of the vegetation characteristics through the GNSS reflections, as, for instance, in [33] and later in [39]. Similarly, the inhabited zones, including buildings, roads or bridges, give a different reflection depending on the surface composition, roughness and inclination. However, the detection of these kinds of targets is not the focus of this work.

The prototype was used in the advanced mode, as detailed in Section 3.3, collecting data from both the RHCP and the LHCP channels of the nadir-pointing antenna. The collected signals were then processed to get the aircraft and the satellite position and to compute the specular points for each satellite. Then, the direct and reflected SNR were estimated from both the RH and the LH circular polarizations, so as to enable the post-processing algorithms presented in Section 4.1 and Section 4.2. It is important to highlight that the data post-processing necessary to convert the reflectometry measurements in Equation (5) or Equation (10) in estimates of the soil moisture is highly sensitive to the accuracy of either the terrain composition model or the terrain scattering model used in the conversion process, as well as to their sensitivity to the signal incidence angle (satellite geometry). Even if the focus of this paper is on the prototype design, both of the hardware and software parts, some results are presented here of the soil parameter retrieval process, in order to validate the system in terms of the final output.

**Figure 10:**
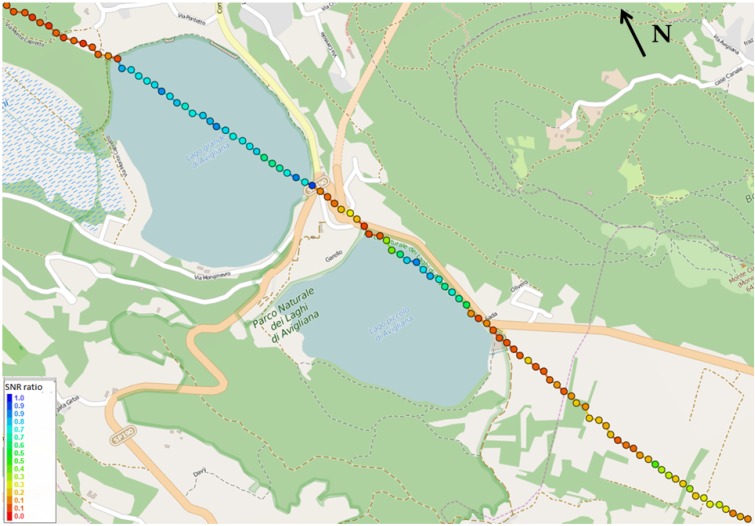
Specular points of PRN 30 over the Avigliana lakes. The color is proportional to the SNR ratio between the left-hand circular polarized (LHCP) reflection and the direct GPS signal.

**Figure 11:**
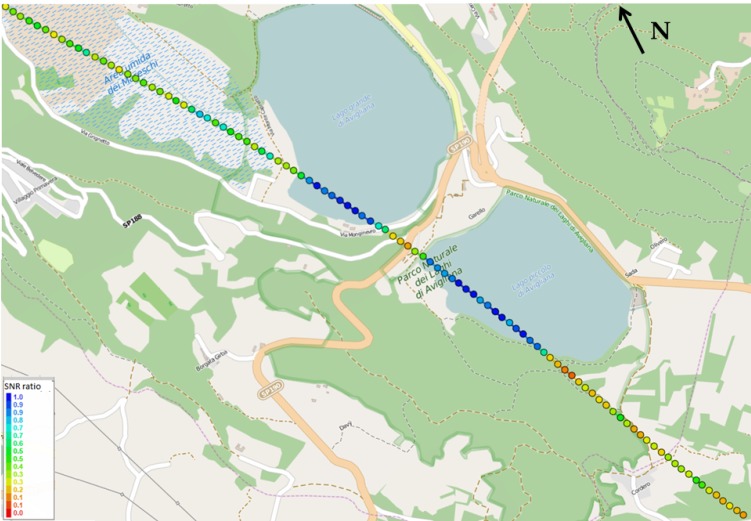
Specular points of PRN 13 over the Avigliana lakes. The color is proportional to the SNR ratio between the LHCP reflection and the direct GPS signal.

Figure 10 and Figure 11 show the plot on the map of the estimated specular points for two satellites (PRN30 and PRN 13, respectively); the color is proportional to the ratio between the reflected LHCP SNR and the direct GPS signal SNR, which is related to the geophysical quantity of interest, the soil dielectric constant, through Equations (3) and (4). As explained above, if a suitable model of the soil composition is given, then the full soil moisture information can be retrieved through the measurement of the LHCP reflections only, assuming the approximation of a smooth surface.

As expected and indicated by the red points in Figure 10 and Figure 11, the reflection from the water surface is much higher than from the terrain [34], where weaker and different values correspond to different lands, such as forest or fields, with different moisture levels. Furthermore, the strength of the LHCP reflections is more intense as the satellite elevation increases. In this case, while the elevation of PRN 13 is around 45${}^{\circ }$, PRN 30 has an elevation lower than 10${}^{\circ }$, showing reduced power values on the reflected signal.

Quantitative measurements validate this expectation: on the water, the SNR ratio assumes values between 0.7 and 1 (the differences are mainly due to the elevation angle and the noise effects), whereas when the specular point is on the terrain, the measured values are very different. Figure 10 shows that for PRN 30, the reflection points out of water are mainly on a forest area (northern points) and on irrigated fields (southern points), showing SNR ratios below 0.2 and between 0.2 and 0.4, respectively. As shown in Figure 11, the reflection points for PRN 13 are southern with respect to PRN 30, and they fall also in the so-called “Area umida dei Mareschi”, *i.e.*, the “Mareschi humid zone”. In that region, as expected, the SNR ratios oscillate in a range between 0.45 and 0.65, sometimes very close to the values assumed on water basins, due to the swamp effect. Then, in the southern region of irrigated fields, again, the measurement is similar to PRN 30, with values between 0.2 and 0.45, with small differences due to the satellite elevation and specular point positions.

**Figure 12:**
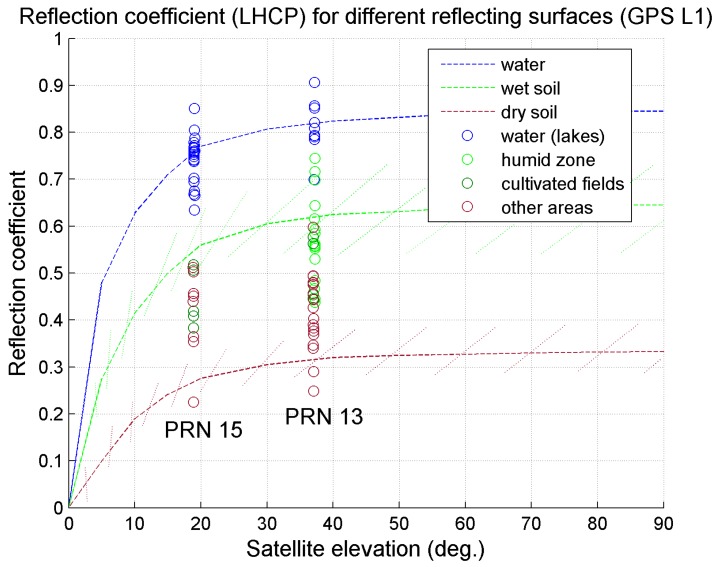
Reflection coefficient: comparison between expected and measured values, for different reflecting surfaces and satellite elevations.

Figure 12 shows a comparison between some in-flight real measurements and the expected values of the reflection coefficient for different satellite elevations, *i.e.*, different incidence angles, and three types of surface: water (blue), wet soil (green) and dry soil (brown). The expected values of the reflection coefficient, represented by dotted lines, are available in the literature [18,40] and, being the result of accurately calibrated test campaigns, have become the theoretical reference for this kind of measurement. Note that in the literature, the qualitative expressions dry soil and wet soil are largely used, to indicate poor and abundant water content in the soil, respectively. Since it is correct to think that the expressions wet and dry soil are related to a range of water content values, in Figure 12, a region of values is indicated for the dry and the wet soil, by the dashed bars, brown and green respectively. The circle dots in the figure indicate the measured values, for PRN 13 and 15 respectively. The measurements, as for Figure 11, are taken at a rate of 1 Hz. From Figure 12, it can be seen how the values obtained on the water surface are different for two satellites at different elevation, as expected, as well as in the other regions. Note that different colors are used to plot the circle dots, depending on the region in which the reflection point lays, known from the map. For instance, for the PRN 13, when the reflection point is in the Mareschi humid zone, the reflection index is plotted using the light green color. As is visible from Figure 12, the reflection coefficient over that region has a value that matches the expected one. However, the variance of the measurements, including those obtained on the water surface, which should be expected to be fairly homogeneous, is due to different factors. First, it has to be noted that, in general, the estimate of the SNR of the direct GPS signal, even in static conditions, has a certain variance, in the order of a few dB-Hz, due to several reasons, including the high noise present in the signal. In this environment, in-flight, more effects contribute to the variance, as, in particular, the antenna gain, not being omnidirectional. The use of different antennas being non-co-located, for the direct and the reflected signal, increases the effects of these phenomena. However, the level of accuracy reached is as expected from the project requirements, given the low-cost devices, which cause several residual errors, not including accurate hardware calibration of the antennas (using inertial systems) and not calibrating other effects, such as the system vibrations.

A further test was done by comparing the retrieved values of soil moisture, for the same area, computed using the reflections from different satellites. This test can be a good proof of the goodness of the system, allowing a comparison between different measurements (signals from different satellites) of the same quantities (same area). This was possible thanks to the multiple passing of the aircraft close to the same area during the flight test. In this way, it happens that different reflections, corresponding to different satellites and then to different incidence angles and to different reflection coefficients, must theoretically give the same result in terms of dielectric constant and moisture, since the reflection points lay in the same area. Applying the soil moisture retrieval algorithm as explained in Section 4.1 to the real in-flight measurements, it is found that when the reflection point is on the lakes’ surface, the average estimated value of the so-called volumetric soil moisture, *i.e.*, the estimate of the soil water content, is as expected $m_{v}≃1$ for all of the PRNs, 13, 15 and 30. Over other regions, for instance cultivated fields, when the reflection points of different satellites lay on the same field (visible from the map, with the satellite view), the mean value of the estimated soil parameters match for different satellites. In the Mareschi humid zone, for instance, the measured mean value is $m_{v}=0.75$ for PRN 13, while it is $m_{v}=0.78$ for PRN 15. The matching between these measurements, together with the comparison between the expected and the obtained measured reflection coefficients in Figure 12, represents a good test of the measurement system, when *in situ* measurements with other sensors are not available.

As said before, the results described above are obtained from the processing of the LHCP reflection. Although it is expected that the processing of the RHCP measurement can improve the accuracy of the overall results, as discussed in Section 4.2, an insufficient cross-polarization separation at the antenna stage is likely responsible for the little improvement observed in our data collections. For this reason, we limit the present discussion to the results of the LHCP-only approach mentioned in Section 4.1. Nonetheless, this test proved the prototype to be effective in order to provide GPS reflection measurements useful to retrieve soil parameters, such as its moisture. The overall accuracy of the methodology depends on several parameters, including the antenna performance, particularly in terms of cross-polarization separation, the applicability of the model used to estimate the soil parameters and the accuracy of the knowledge of the terrain composition.

## 6. Conclusions

This paper presents the design and development of a GNSS passive radar for the classification of lands, based on the water content feature, and the detection of water surfaces’ extent and scattering objects on the ground. Such GNSS passive radar is intended for small UAVs; therefore, size and weight constrained the design of the whole system from the beginning. The sensor features four synchronized RF channels, which are used to receive the direct and the reflected GNSS signals separately over RHCP and LHCP polarizations. The RF part is connected to a commercial embedded micro-processor, which hosts the software routines to control the flow of the digital samples of all channels. The sensor guarantees the storage of more than 30 min of data, if the sampling frequency of the signals is set to 13 MHz. Although the sensor has been integrated with low-cost COTS components, the design followed the software radio paradigm and, for this reason, allows for a significant level of flexibility of the system settings, e.g., the possibility to use only a subset of the four channels, custom frequency plan and variable bandwidths. The sensor has been intensively tested in-lab and validated through some flight tests. These served to assess the performance in a real environment, including the electromagnetic compatibility with other UAV devices, the sensor reliability to store data in an automatic fashion and the mechanical resistance of the sensor’s case during take-off and landing stress. The sensor successfully demonstrated its ability to receive reflected signals, both LHC and RHC polarized. This result is comparable to others presented in the literature, but on the one hand, it allows the simultaneous grabbing of RHCP and LHCP reflections and, on the other hand, has been obtained with a prototype much lighter and smaller with respect to those used in other experiments.

Among all of the results, it is important to underline the lesson learned from the analysis performed over some of the collected datasets. The cross-polarization isolation between the RHCP and LHCP channels of the antenna pointing at the nadir is critical for the system performance. In fact, if the cross-polarization rejection is lower than the minimum required, a portion of the LHCP power obscures the RHCP reflected signals, which cannot be correctly measured. This limits the fine computation of correct soil moisture parameters and identifies the nadir-pointing dual-polarization antenna as the most critical system element, as it requires very high cross-polarization isolation, typically unavailable as COTS.

Furthermore, considering the extreme weakness of the reflected signals, another critical point is the accurate characterization, calibration and control of the electromagnetic environment during the tests and on-board the aircraft during the data collections: the effect of the electromagnetic interference from the surrounding electronic systems during the data collections, especially on-board unmanned vehicles, can be destructive for the GNSS-R processing and, therefore, must be carefully controlled.
